# Elder abuse and hospitalization in rural Malaysia

**DOI:** 10.1371/journal.pone.0270163

**Published:** 2022-06-24

**Authors:** Muhammad Abbas M. Firdaus, Raudah Mohd Yunus, Noran Naqiah Hairi, Wan Yuen Choo, Farizah Hairi, Leny Suzana Suddin, Rajini Sooryanarayana, Norliana Ismail, Devi Peramalah, Zainudin M. Ali, Sharifah N. Ahmad, Inayah A. Razak, Sajaratulnisah Othman, Awang Bulgiba

**Affiliations:** 1 Faculty of Medicine, Universiti Teknologi MARA (UiTM), Sungai Buloh Campus, Sungai Buloh, Malaysia; 2 Department of Public Health Medicine, Faculty of Medicine, Universiti Teknologi MARA, Sungai Buloh, Malaysia; 3 Department of Social and Preventive Medicine, Centre for Epidemiology and Evidence-Based Practice, University of Malaya, Kuala Lumpur, Malaysia; 4 Health and Well-being Research Cluster, Institute of Research Management and Monitoring, Research Management and Innovation Complex, University of Malaya, Kuala Lumpur, Malaysia; 5 Faculty of Public Health, Universitas Airlangga, Surabaya, Indonesia; 6 Department of Social and Preventive Medicine, Faculty of Medicine, University of Malaya, Kuala Lumpur, Malaysia; 7 Family Health Development Division, Ministry of Health Malaysia, Putrajaya, Malaysia; 8 Disease Control Division, Tobacco Control Unit, Ministry of Health Malaysia, Putrajaya, Malaysia; 9 Negeri Sembilan Health State Department, Seremban, Negeri Sembilan, Malaysia; 10 Department of Primary Care Medicine, University of Malaya, Kuala Lumpur, Malaysia; The University of Mississippi Medical Center, UNITED STATES

## Abstract

Our study aims to describe and determine factors associated with hospitalization among victims of elder abuse and neglect (EAN) in rural Malaysia. A cross sectional study based on the baseline data of the Malaysian Elder Mistreatment Project (MAESTRO) collected from November 2013 until July 2014 involving 1927 older adults in Kuala Pilah, Negeri Sembilan was conducted. EAN was determined using the modified Conflict Tactics Scale (CTS) and hospitalization rates were determined based on self-report. The prevalence of overall EAN was 8.1% (95%CI 6.9–9.3). Among male respondents, 9.5% revealed history of abuse and among female respondents, 7.2% reported experiencing EAN. The annual hospitalization rates per 100 persons within the past one year among EAN victims and non-victims were 18 per 100 persons (SD = 46.1) and 15 per 100 persons (SD = 64.1) respectively. Among respondents with history of EAN, 16.0% (n = 21) had been hospitalized in the past 12 months while among respondents with no EAN experience, 10.2% (n = 153) were hospitalized. Multivariable analyses using Poisson regression did not show any significant association between EAN and hospitalization. This could be due to the complex interactions between medical and social circumstances that play a role in hospital admissions, factors affecting the health care system, and access to health care among EAN victims.

## Introduction

The World Health Organization (WHO) has recognized elder abuse and neglect (EAN) as a worldwide underreported public health issue [1]. It is not a novel phenomenon and has existed in the community since antiquity [2]. According to literature, EAN is a frequent occurrence, costly, predictable and may cause mortality in certain occasions [3]. Various definitions of EAN persist around the globe even though there are some similarities among the definitions. Even within the same country, the definition of EAN and its subtypes differ according to the source, author or agencies [4]. The WHO defined EAN as ‘a single or repeated act, or lack of appropriate action, occurring within any relationship where there is an expectation of trust, which causes harm or distress to an older person’ [1]. There are five subtypes of EAN namely physical, financial, sexual, psychological and neglect [5]. The prevalence of EAN varies according to demographic, setting, terminology and research methodology used [3]. Global EAN prevalence was reported as ranging between 3.2% and 27.5% [6], with the prevalence in developing countries ranging from 13.5% to 28.8% [7]. In another study, the global pooled prevalence was 15.7% [8]. On the other hand, the 2018 National Health Morbidity Survey (NHMS) reported that 9.0% older respondents experienced EAN, while two local studies in Kuala Pilah found a prevalence of 8.1% and 4.5% since the age of 60 and in the past 12 months, respectively [9–11]. The prevalence of EAN from these studies differ as there are differences in their study design and study population; some studies exclude certain populations with higher risk of abuse such as older population with dementia illness [12]. Underreporting of EAN is occurring globally, even in regions where reporting is required by the law [13].

There is scarce evidence on the impact of EAN as compared to other types of interpersonal violence. The impact of elder abuse may be direct or indirect. The health consequences of EAN demonstrated in empirical studies can be categorized into mortality, morbidity and healthcare utilization. Strong evidence shows that mortality outcome is greater in abused older victims as compared to their counterparts [14]. It’s impact on victims psychological or emotional well-being makes them more likely to have anxiety and stress, symptoms of depression, suicidal ideation and sleeping disorders [15–17]. They also have higher chances of experiencing chronic pain, metabolic syndrome, gastrointestinal disturbance symptoms and disability [18–21].

Healthcare utilization involves emergency department (ED) visits, outpatient department visits and hospitalization. Current evidence shows that abuse victims have higher rates of utilisation and hospitalisation as compared to their non-abused counterparts [22–25]. The direct yearly medical costs attributable to the injuries secondary to EAN were estimated at $5.3 billion in the United States alone [26]. In Australia, hospital admission cost due to EAN were calculated to be between AUD 9.9 million and AUD 30.7 million in year 2007–2008 [27]. However, empirical studies that link EAN and health care utilization have been derived largely from high-income countries, such as the United States and Sweden. Data are sparse for developing countries. The pattern or trend of healthcare utilization in the context of EAN might be different in low- and middle-income regions given the distinct structure and organization of the healthcare system and social services.

EAN is largely considered a taboo subject in Asian society. It is not openly talked about due to the culture of filial piety (respect and care for elders) [28]. Furthermore, the elder populations are frequently seen as a source of wisdom and experience [29]. This leads to the subject being little explored and understood. The research gap identified was the scarcity of studies analysing health care utilization–including hospitalization–as an outcome among EAN victims. All the studies examining healthcare utilization or hospitalization were from high income countries. So, this study aims to fill the research gap by examining the relationship between EAN and hospitalization in the Malaysian context. Our study aims to: (1) compare the frequency of hospitalization in the past 12 months between EAN victims and of non-victims and (2) determine the association between EAN status and hospitalization rates. To our best knowledge, this is the first study that investigates hospitalization rates among abused rural elders in the context of Southeast Asia.

## Methods

### Study design

This was a cross sectional study based on the secondary data of the Malaysian Elder Mistreatment Project (MAESTRO) using the baseline data collected from November 2013 until July 2014 [30]. It was a community-based prospective cohort study. The purpose of this project was to describe the distribution of elder mistreatment, determine the epidemiological characteristics and evaluate the consequences of elder abuse. This project involved multiple waves of data collection with the baseline data collected from November 2013 until July 2014.

### Setting

The study took place in Kuala Pilah District, a largely rural area in the Malaysian state of Negeri Sembilan.

### Sampling method

We used universal sampling of all participants in the secondary data of the MAESTRO project, which involved 1927 participants. However, for this analysis, 294 participants were removed due to incomplete data on healthcare utilization and severe cognitive impairment. In the primary MAESTRO project study, the samples were obtained through a multistage sampling strategy using the sampling frame prepared by Department of Statistics Malaysia established from the 2010 national census conducted. The study managed to recruit 1927 individuals aged 60 years and more. Respondents were interviewed at home by trained personnel who administered standardized questionnaires which had been validated [30]. Each home had one elder person and one caregiver interviewed.

Inclusion criteria were all participants within the MAESTRO dataset aged 60 years and above living in the Kuala Pilah District that had completed the modified Conflict Tactic Scale (CTS) questionnaire. The exclusion criteria were incomplete information (missing data) in the study data on history of hospitalization and those with severe cognitive impairment based on Mini Mental State Examination (MMSE) scores. Severe cognitive impairment was defined as having an MMSE score of less than ten [31].

### Measures

The outcome variable in this study was frequency/rates of hospitalization in the past 12 months. The sociodemographic variables included in this study were age, sex, education level, household income, and ethnicity. The health-related variables included comorbidities and self-rated health. Psychosocial variables were depression and social support. The cognitive status variable was also included in this study.

### Elder abuse and neglect

In this study, elder abuse and neglect is the independent variable. The operational definition used was any abuse and neglect that occurred to the victim since the age of 60-years, or also known as lifetime EAN. This definition is according to research done in Ireland with some adjustment of the duration, taking into account the experience beginning since age 60 years instead of within the past 12 months among those aged more than 60 years [32]. Operational definitions of physical, financial, or sexual abuse was at least one single episode of abuse reported by the elder respondent committed by a trusted person that includes neighbours, family members or friends. Operational definitions of psychological abuse and neglect are at least 10 episodes in a duration of one year since age 60 years perpetrated by someone in a position of trust as stated by the elder participant. Even though there were less than ten such instances in a year, psychological abuse and neglect were still considered if the episode(s) was regarded as having a severe impact by the older adult. The questions to evaluate the neglect component were based on two tools that is The Katz Activities of Daily Living (Katz ADL) and Lawton Instrumental Activities of Daily Living Scale (Lawton IADL). Both of these tools had been validated [33].

### Hospitalization

Hospitalization was defined as history of being admitted to hospital for at least twenty-four hours within the last one year. The respondents were asked regarding history of being admitted to the hospital and if the answer was yes, the subsequent question asked was the number of times. This variable was treated as both categorical (yes/ no) and continuous variable (frequency) throughout the various stages of analyses.

### Covariates

Sociodemographic variables in this study were age, sex, ethnicity, household income and education level. Income was grouped into “Low” (monthly household income of less than RM1000 per month), “Medium” (monthly household income of RM1000 –RM2499 per month) and “High” (monthly household income of RM2500 and more). Education level was categorized into “Low” (no formal education), “Medium” (entered elementary or high school) and “High” (entered college or university).

Health-related variables measured were self-rated health and comorbidities. Self-rated health was determined by asking the respondent “How do you rate your health?”. Response options were: (1) “Very poor”, (2) “Poor”, (3) “Good” and (4) “Very good”. The details of comorbidities were based on self-reported data that includes (1) diabetes, (2) hypertension, (3) hypercholesterolemia, (4) any types of cancer, (5) arthritis, (6) coronary heart disease, (7) stroke and (8) congestive heart failure.

Psychosocial variables included were depression, social support and cognitive impairment. Depression was measured using the 15-item Geriatric Depression Scale. Scores of zero to four indicated no depression (normal), five to nine indicated mild depression and ten or more indicated severe depression. The Duke Social Support Index (DSSI) was used to measure social support, and higher scores indicated higher levels of social support. In this study patient with severe cognitive impairment were excluded from the sample using Mini Mental State Examination (MMSE).

### Analytical approach

The IBM Statistical Package for Social Science (SPSS) software version 26.0 for Windows was used to analyse the data. For descriptive statistical analyses, categorical variables were reported in frequencies and percentages, while continuous variables were reported in means and standard deviations. The analysis of association between categorical variables were done using Pearson’s Chi Squared test or Fisher’s Exact test if indicated. The Independent t-test was used to compare the means of two independent groups. Poisson Regression analysis was done to determine the association between EAN and hospitalization rates. The Poisson model is a useful methodology for count data, such as the yearly rate of hospitalization. Poisson regression is widely used to evaluate the effect of various treatments or risk factors on hospitalization rates [34–37]. All the assumptions of selected statistical tests were checked prior to running the analyses and the statistical significance value was set at 0.05.

All the five assumptions of Poisson analysis were checked one by one prior to analysis [38]. First, the dependent variable must consist of count data, which was annualized hospitalization rate in this study. Second, the independent variables must consist of continuous and categorical variables as explained in the methodology section. Third, independence of observations, which means that the measurements for each sample subject are in no way influenced by or related to the measurements of other subjects. This is considered to have been met, given the random sampling approach explained before. Fourth, the distribution of counts must follow a Poisson distribution. The Poisson distribution was tested using the One-Sample Kolmogorov-Smirnov Test, where the mean for Poisson Parameter was 0.15 and the p-value was not significant at 0.06. This means that the dependent variable (number of hospitalizations within past one year) was following the Poisson distribution. Fifth, the mean and variance of the model should be identical. In this model, the ratio between deviance and degree of freedom (*df*) value from the Goodness-of-fit table was less than one, at 0.63. There was no overdispersion problem and thus this model was deemed appropriate.

### Secondary data cleaning

**Fig 1:**
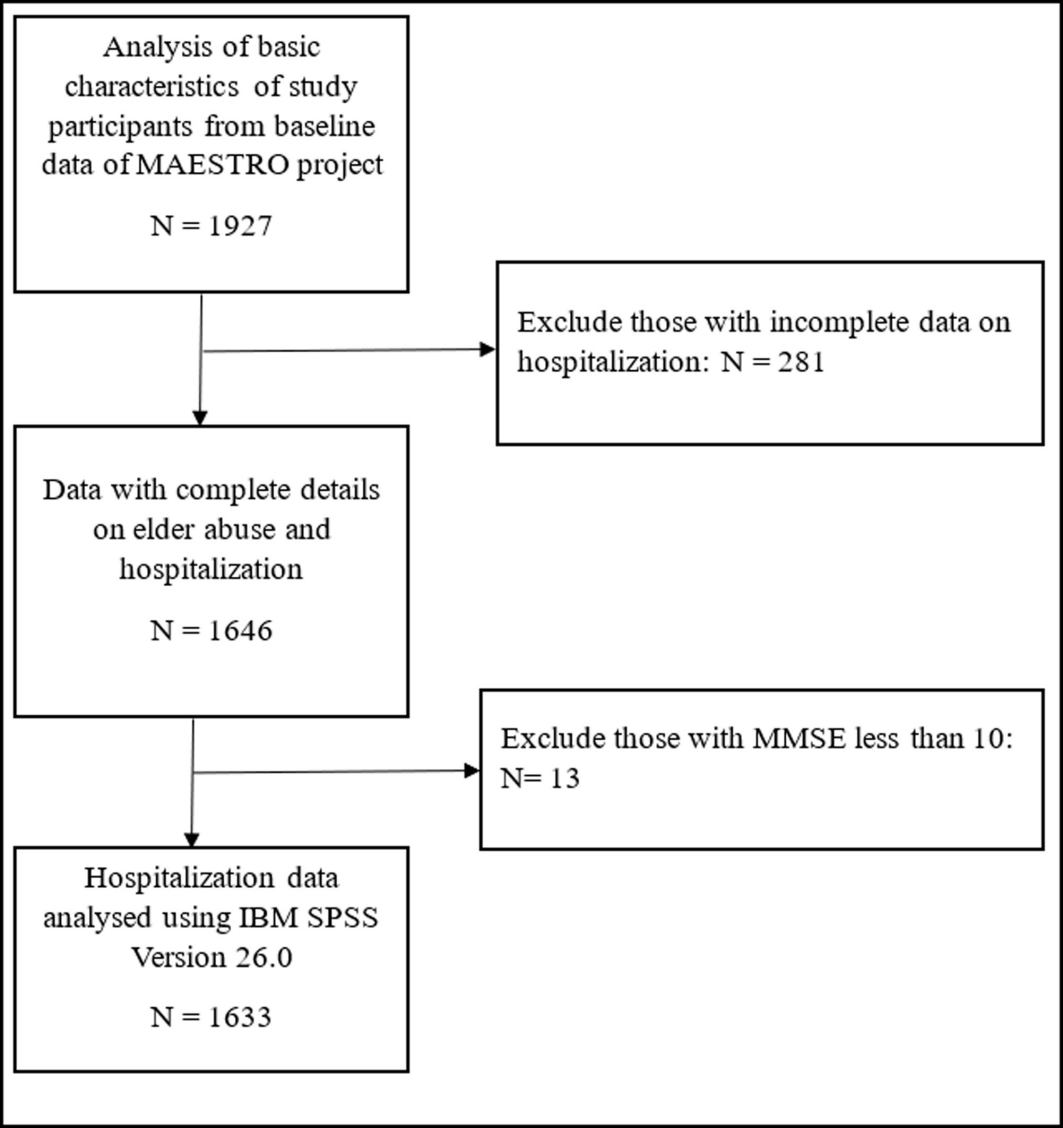
Data management flow.

The total number of datasets received from the data custodian was 1927 datasets for the baseline data. A total of 281 records (14.6%) from the dataset were excluded due to incomplete information on hospitalization and another 13 (0.7%) were excluded due to MMSE score being less than 10. The final number of records for analyses were 1633. The data management flow is as in Fig 1. The other variables were checked for missing data. Missing completely at random (MCAR) was determined using Little’s test. Little’s test for all the variables showed a p-value of more than 0.05, which concludes the data is missing completely at random. Since the range of missingness percentage is between 0.1–2.0% which is less than 5% for all items, it was assumed that this would not cause any substantial bias to the results [39]. Fig 1 summarizes the data management flow.

### Ethics

For this study, ethical exemption was given by the Research Ethics Committee Universiti Teknologi Mara (UiTM), as secondary data was used (Reference number: REC/07/2021 (EX/106)). The data were analysed using the unique ID numbers as identifiers, without the individuals’ personal information, as this study involved the usage of secondary data. No contact was made with the respondent for this secondary data study. The primary MAESTRO study was given ethical approval by Medical Ethics Committee of the University of Malaya Medical Centre (MEC Ref 902.2) and Malaysian National Medical Research Register (NMRR-12-1444-11726). In the primary MAESTRO study, written informed consent was obtained from all the participants. The objective, risks, and advantages were described to the participants. The participation was voluntary, and they could withdraw or opt out at any time without compromising their rights to medical or social welfare services [30].

## Results

In this study, females formed 60.8% (n = 1172) of total respondents. The mean age was 69.8 years (SD = 6.9). Meanwhile, the mean age of abuse victims was 70.0 years (SD = 6.7) and for those not abused, 69.7 years (SD = 7.0). Females comprised 53.8% (n = 84) of total respondents in the abused category. Malays were the form the major ethnic group at 96.9% (n = 1868). As this study was done in a rural area, almost all (97.7%) of the respondents studied up to secondary school and 96.4% had monthly household income less than RM2500. The sociodemographic characteristics of our study cohort are shown in Table 1.

**Table 1:** Basic characteristics of study participants (N = 1927).

Variable	All, n (%)	Abused, n (%)	Not abused, n (%)	P-values
**Age Group**				
60–69	981 (50.9)	80 (51.3)	901 (50.9)	0.858^a^
70–79	769 (39.9)	60 (38.4)	709 (40.0)	
80 and above	177 (9.2)	16 (10.3)	161 (9.1)	
**Sex**				
Male	755 (39.2)	72 (46.2)	683 (38.6)	0.063 ^a^
Female	1172 (60.8)	84 (53.8)	1088 (61.4)	
**Ethnicity**				
Malay	1868 (96.9)	149 (95.5)	1719 (97.1)	0.290 ^a^
Chinese	21 (1.1)	2 (1.3)	19 (1.1)	
Indian	30 (1.6)	5 (3.2)	25 (1.4)	
Others	8 (0.4)	0 (0.0)	8 (0.4)	
**Education level**				
Low	238 (12.4)	13 (8.4)	225 (12.8)	0.262 ^a^
Medium	1638 (85.3)	139 (89.7)	1496 (84.9)	
High	44 (2.3)	3 (1.9)	41 (2.3)	
**Household income**				
Low	1244 (64.6)	117 (75.0)	1127 (63.6)	**0.007** ^a^
Middle	612 (31.7)	32 (20.5)	580 (32.8)	
High	71 (3.7)	7 (4.5)	64 (3.6)	
**Chronic disease**				
Diabetes mellitus	531 (27.9)	50 (32.3)	481 (27.5)	0.209 ^a^
Hypertension	1034 (53.8)	81 (51.9)	953 (54.0)	0.618 ^a^
Hypercholesterolemia	664 (34.6)	49 (31.4)	615 (34.9)	0.385 ^a^
Coronary heart disease	127 (6.6)	13 (8.4)	114 (6.5)	0.354 ^a^
Stroke	46 (2.4)	6 (3.9)	40 (2.3)	0.214 ^a^
Arthritis	387 (20.2)	38 (24.5)	349 (19.8)	0.165 ^a^
Cancer	17 (0.9)	0 (0.0)	17 (1.0)	0.220 ^a^
Congestive heart failure	46 (2.4)	4 (2.6)	42 (2.4)	0.869 ^a^
**Self-Rated Health**				
Poor	535 (27.8)	31 (32.7)	484 (27.4)	0.159 ^a^
Good	1389 (72.2)	105 (67.3)	1281 (72.6)	
	Mean (SD)	Mean (SD)	Mean (SD)	
**No. of co-morbidities**	1.5 (1.3)	1.6 (1.4)	1.5 (1.3)	0.537 ^b^
**Depression (GDS)**	3.9 (3.7)	4.8(3.8)	3.9(3.7)	**0.003** ^b^
**Social support (DUKE)**	27.4(3.3)	26.8(4.1)	27.4(3.1)	0.020 ^b^

Bolded values are statistically significant at p<0.05.

^a^ Chi-square p-value.

^b^ Independent T-test p-value.

A total of respondents (156) had reported having experienced abuse since the age of 60-years, year leading to a prevalence of EAN of 8.1% (95% CI 6.9–9.3) in this study. The commonest subtype of abuse was financial abuse at 4.8% (n = 92), followed by psychological abuse 3.4% (n = 65). Next was physical abuse at 1.2% (n = 24), neglect 1.1% (n = 22) and the least common subtype was sexual abuse at 0.3% (n = 5). With regards to age group, 7.6% (n = 65) of those aged 60–69 years reported having abused, while 8.2% (n = 52) and 9.3% (n = 14) reported EAN experiences among those aged 70–79 years and 80 years and older, respectively. This upward trend showed a dose-response relationship between the proportion of EAN victims and age; the higher the age group, the higher the percentage of respondents reporting EAN. Among male respondents, 9.5% (n = 72) revealed a history of abuse and 7.2% (n = 84) among female respondents reported the same.

Among respondents with a history of EAN, 16.0% (n = 21) had been hospitalized in the past 12 months; meanwhile among the respondents without EAN 10.2% (n = 153) were hospitalized. This is illustrated in Table 2.

**Table 2:** Hospital admission between those abused and not abused (N = 1633).

Variables	Abused	Not abused	P-value
**Hospitalization within past 12 months, n (%)**			
Yes	21 (16.0)	153 (10.2)	**0.038** ^a^
No	110 (84.0)	1349 (89.8)	
**Rate of hospitalization, mean, per 100 persons (SD)**	18 (46.1)	15 (64.1)	0.514 ^b^

Bolded values are statistically significant at p<0.05.

^a^ Chi-square p-value.

^b^ Independent T-test p-value.

Examining each factor individually, older adults aged 80 years and more, female sex, those with poor self-rated health, more chronic diseases and depression were all found to have significant associations with hospitalization rates using Poisson regression analysis. However, there was no significant association between EAN and hospitalization. This is illustrated in Table 3.

**Table 3:** Univariable regression table to show the association between EAN status and hospitalization.

Variable	Crude Incidence Rate Ratio^a^	95% CI	Standard error	P-value
**Established EAN Status**	1.26	0.82–1.92	0.22	0.288
**Age (reference 60–69 years)**				
**70–79**	1.22	0.93–1.59	0.14	0.158
**80 and more**	1.82	1.23–2.68	0.20	**0.003**
**Sex (reference male)**				
**Female**	0.59	0.46–0.76	0.13	**<0.001**
**Ethnicity (reference Malay)**				
**Non-Malay**	1.80	0.96–3.39	0.32	0.068
**Education level (reference high)**				
**Low**	2.59	0.62–10.84	0.73	0.192
**Medium**	2.47	0.61–9.95	0.71	0.203
**Household income (reference high)**				
**Low**	1.68	0.74–3.78	0.41	0.212
**Medium**	0.84	0.36–1.97	0.43	0.695
**Self-rated health (reference good)**				
**Poor**	2.84	2.21–3.65	0.13	**<0.001**
**Number of Chronic diseases**	1.43	1.31–1.55	0.04	**<0.001**
**Depression (GDS)**	1.14	1.08–1.19	0.02	**<0.001**
**Social support (DUKE)**	0.99	0.96–1.03	0.02	0.892

Bolded values are statistically significant at p<0.05.

^a^ Poisson Regression Analysis.

In the multivariable analyses the incidence rate ratio (IRR) was found to be significant for sex, number of chronic diseases, depression, and self-rated health. The IRR for hospitalization was lower among females compared to males (0.58, 95% CI: 0.44,0.77) and higher for those with more chronic diseases (1.33, 95% CI: 1.21,1.45), higher scores of GDS for depression (1.06, 95% CI 1.00–1.12) and older adults who rated their health poorly (2.18, 95% CI: 1.66, 2.86). Table 4 demonstrates the relationship between the different variables–including EAN–and hospitalization using Poisson regression.

**Table 4:** Multivariable regression table to show the association between EAN status and hospitalization.

Variable	Adjusted Incidence Rate Ratio ^a^	95% CI	Standard error	P-value
**Established EAN Status**	1.03	0.67–1.59	0.22	0.878
**Age (reference 60–69 years)**				
**70–79**	0.95	0.72–1.26	0.14	0.731
**80 and more**	1.22	0.81–1.83	0.21	0.352
**Sex (reference male)**				
**Female**	0.55	0.42–0.72	0.14	**<0.001**
**Ethnicity (reference Malay)**				
**Non-Malay**	1.51	0.79–2.89	0.33	0.213
**Education level (reference high)**				
**Low**	1.71	0.38–7.62	0.76	0.480
**Medium**	1.51	0.36–6.4	0.73	0.569
**Household income (reference high)**				
**Low**	1.47	0.63–3.41	0.43	0.371
**Medium**	0.78	0.33–1.87	0.44	0.582
**Self-rated health (reference good)**				
**Poor**	2.11	1.60–2.79	0.14	**<0.001**
**Number of Chronic diseases**	1.33	1.21–1.45	0.46	**<0.001**
**Depression (GDS)**	1.06	1.00–1.12	0.03	**0.041**
**Social support (DUKE)**	1.02	0.98–1.07	0.22	0.312

Bolded values are statistically significant at p<0.05.

^a^ Poisson Regression Analysis.

Based on descriptive statistics, the percentage of hospitalization and the mean rate of hospitalization was higher among the EAN victims as compared to non-victims. Unadjusted analysis shows that hospitalization was significantly associated with age (80 years and more), sex (being female), poor self-rated health, depression and number of co-morbidities. However, EAN was not significantly associated with hospitalization based on Poisson regression analysis after adjusting for all the other variables.

## Discussion

In this study involving 1927 participants, the prevalence of lifetime EAN stood at 8.1%. The overall annual rate of hospitalization of EAN victims was higher than those without similar history. Annualized hospital admission rates were found to be higher among the abused elders as compared to non-abused counterparts (18 per 100 persons in abused elders vs 15 per 100 persons in non-abused elders). There is limited literature reporting on EAN in the community-dwelling population and hospitalization rate. In one study by Dong et al. (2013), there was significant difference of hospitalization rate among reported elder abuse victims (1.97) and non-victims (0.62). Contrary to the existing evidence that corroborates the relationship between EAN and health care utilization, this study did not find a similar link between EAN & hospitalization. We offer some possible explanation here.

Hospitalization is a complex interaction between medical and social circumstances, especially among older adults, and it is more than a simple medical event [40]. The requirement for hospitalization varies greatly depending on the social condition of the patients. An elder with good social support might not require hospital admission even if they suffer from severe medical conditions, while patients with poor social support or abused victims might need hospitalization for simple exacerbations of their medical condition [40]. However, another study found that older individuals who report having such support are somewhat more likely to have been hospitalised in the previous year than those who do not receive such support [41]. This conflicting finding is not uncommon when discussing the association between EAN and healthcare utilization [22]. There is incomplete understanding of health care utilization among those who are victimized as prior case reports indicate that elder abuse victims utilise health care regularly. Nevertheless, recent epidemiological research has produced contradictory results in health service usage among individuals reported to be abused [23].

Healthcare utilization can be categorized into outpatient or primary care utilization, emergency department visits, and hospitalization. It is generally hypothesised that EAN has a different impact on several elements of healthcare utilisation. The underlying issues that may affect healthcare utilization among EAN victims include, for instance, perpetrators not providing adequate support and care, perpetrators isolating the victims, abuse-related health issues requiring urgent intervention, victims having poor health-maintenance behaviour including medication non-adherence, substance abuse and poor health literacy [42]. These factors lead to EAN victims having higher rates of utilization of the emergency department, admission to the hospital and placement in nursing home [22–24, 43, 44]. The similar underlying issues can also cause reduced utilization of outpatient and primary health care including lesser primary care visits, more primary care provider changes, bad continuity of care and medications are not refilled when necessary [42]. This poor utilization of primary care will then lead to frequent different emergency departments visits to avoid abuse detection [45, 46]. The average yearly rate of ED visits for older persons who were known victims of elder abuse in the community was 2.11, compared to 0.74 visits per patient per year for older adults who had never been abused [20]. At the same time, abuse was linked to a lengthier duration of stay in the hospital [47, 48].

The rate of health consumption and access to healthcare services can be affected by the healthcare system in a country. In general, middle- and low-income countries have less sophisticated health systems and lack comprehensive social services that detect abuse victims and facilitate their access to physicians or health facilities [49]. In addition, access to health care services (including hospitalization) can be affected by availability of insurance coverage and where medical services are free (or subsidized), other factors can still hinder access such as transportation costs. Studies have also showed that health care utilization is influenced by health-seeking behavior, and the latter in turn is influenced by sociocultural norms [50–52]. For example, in Malaysia it is still relatively common for people to seek traditional medicine or consult unauthorized traditional healers instead of doctors, especially when facing complex health issues [53, 54]. Lastly, hospital admission is more likely to be caused by physical abuse, a subtype of EAN that often manifests in the forms of physical signs and injuries. The other EAN subtypes are more subtle–and may take a long time and very severe forms–before victims need hospitalization. In this study, the prevalence of physical abuse was 1.2% which came third after financial and psychological abuse. Non-physical health impact such as depression and poor nutrition can be treated as outpatient unless they occur in severe forms. Therefore, the lack of association between hospitalization and EAN could have been influenced by this low prevalence.

Evidence that corroborates higher risk of hospitalization among EAN victims is largely derived from the United States. It is known that the Adult Protection Services (APS) in the US employs trained personnel who actively look for suspected abused victims and refer them to health personnel or health facilities [55]. This could have made hospital admission easier as victims are actively detected, and cases investigated and followed-up. A similar support system may not exist across developing regions or when it does, the extent and nature or services can be different. For example, EAN cases largely fall under the jurisdiction of the Social Welfare Department (JKM) in Malaysia. However, JKM does not have a specific team or unit that specializes in the active detection of EAN cases and linking of victims to health services.

Of equal importance is to note that the lack of association in this study did not necessarily mean that abused victims did not use healthcare facilities more frequently compared to non-victims Our study only examined hospitalization, which is one component of health care utilization. We did not include data related to outpatient and ED visits among respondents. It was possible that victims visited the ED or primary care facilities more often in the past year (but did not get hospitalized), as hospitalization is usually reserved for severe cases. As comparison, in child abuse cases, admission is done to ensure the child’s safety even when the health condition is not as severe since this is stated in the law [56]. However, in EAN, admission is most often based on the health condition of the patient. Besides, the lack of association could mean abuse victims had less access to health care due to the social isolation, deprivation and control by perpetrators.

In a nutshell, our findings are not in line with prior evidence on the relationship between EAN and hospitalization rates. This is a new and important contribution to the existing body of knowledge as our results suggest that the relationship between EAN and hospitalization is not linear or straightforward. Rather, it is complex and can be influenced by various factors. These factors include–but are not restricted to–the healthcare system, availability of welfare or protective services that detect EAN, socio-cultural norms that affect health-seeking behavior, and access to health care. Our findings also indicate a possible ‘gap’ or unmet health needs of EAN victims in low-resource settings, while highlighting the crucial role of healthcare providers in EAN detection.

### Study limitation

The interpretation of this study findings should be done in the light of several constraints. First, 281 samples were excluded from the study due to incomplete history of hospitalization. This group could have comprised individuals with different characteristics; they might have poorer health and thus greater hospitalization episodes. Second, our study excluded respondents with severe cognitive impairment. Poor cognition and dementia have been implicated as risk factors for elder abuse [12]. Excluding this group may have underestimated the prevalence of EAN and hospitalization rates. Third, some confounding factors were not included and analyzed in this study, such as those related to the individual (perpetrator), relationship, community and societal factors. Fourth, history of hospitalization was based on self-report so recall bias could not be excluded. However, in the baseline MAESTRO study, the interviewers attempted to verify the information with caregivers or family members. Nevertheless, we are aware that this did not guarantee accuracy of information. Next, given the cross-sectional design, causal relationships between EAN and hospitalization rate could not be ascertained.

### Study strengths

One of the main strengths of this study was the sampling method that ensured a representative sample from the rural area of Kuala Pilah. The sampling frame of the participants is also representative of the elder population in rural Malaysia as it was obtained from the national census. Another strength was that we employed numerous validated tools such as CTS, MMSE, DSSI, and GDS in measuring variables.

## Conclusion

Overall, our study found no statistically significant association between EAN and hospitalization rates after adjusting for potential confounders. This could be attributed to various factors as mentioned earlier. The lack of association however does not deny the higher health needs of EAN victims, as adverse health impacts of abuse in late life have been well-documented. Our findings could also imply an unmet health care need among EAN victims in the rural area. More studies are needed to determine the health care utilization patterns among EAN victims given its implications on health care costs at the individual and national level. In addition, presentation of victims to healthcare facilities provides a window of opportunity for healthcare professionals to screen and intervene. Healthcare professionals should be trained to identify and manage EAN victims encountered in clinical settings, and initiate referrals to other service providers such as social workers. Management of EAN should involve multidisciplinary teams to ensure a holistic approach in catering for victims’ needs and well-being.

Longitudinal, prospective studies are needed to ascertain the causal relationship between various subtypes of EAN with healthcare utilization including hospitalization, and to identify the factors associated with hospitalization in different settings. Healthcare utilization patterns might be different in the urban area where healthcare facilities are more easily accessible compared to the rural setting. As Malaysia is a multiracial country, future studies should attempt to include other races, especially the minority and marginalized groups. Studying institutionalized elders might require a different approach, due to the unique living environment, available services and ecosystem to which residents are exposed in comparison to their community-dwelling counterparts.
